# A new classification allowing assessment of instrumental vaginal-birth practices

**DOI:** 10.1186/s12884-024-06410-5

**Published:** 2024-03-20

**Authors:** Marine Schaeffer, Marie-Caroline Faisant, Alexandre Buisson, Manon Vanneaux, Pascale Hoffmann, Didier Riethmuller

**Affiliations:** 1 Department of Gynecology-Obstetrics and Reproductive Medicine, Grenoble Alpes University Hospital, Grenoble, 38043 France; 2 Gynecology-Obstetrics Department, Annecy Genevois Hospital, Epagny Metz-Tessy, 74370, France

**Keywords:** Instrumental delivery, Classification, Pregnancy, Practice assessment

## Abstract

**Background:**

Instrumental vaginal birth, a very common intervention in obstetrics, concerns nearly one in eight women in France. Instrumentally assisted vaginal childbirth can be for maternal and/or fetal indications. Although it reduces recourse to caesarean section, it is subject to risks. Practices concerning instrumental birth are disparate, varying among different practitioners, maternity units and countries, and it is essential to be able to evaluate them. Our objective was to create a classification tool of women requiring instrumental birth to facilitate the analysis of practices within our maternity unit as well as to enable temporal and geographical comparisons.

**Materials and methods:**

We propose a simple and robust classification based on the same principles as Robson's classification. It is made up of seven totally inclusive and mutually exclusive groups. Our classification was refined and validated using the Delphi method by a panel of 14 experts from throughout France, and tested in our maternity unit using data from throughout 2021.

**Results:**

The seven clinically relevant groups are based on five obstetric criteria (multiplicity, presentation, gestational age, previous type of birth, induction of labor). To classify each woman in a group, five successive questions are posed in a predefined order. The classification has been validated by the experts with highly satisfactory overall agreement.

**Conclusion:**

In order to improve the quality of care, we propose a tool to standardize the evaluation of instrumental vaginal birth practice (called the “Isère classification”, after the county where we work in south-eastern France). It will also facilitate the comparison the practices among different maternity units in a network, a country or even among different countries.

## Background

Instruments such as the ventouse, forceps, or spatulas are often used to assist vaginal birth. The medical indications for instrumental vaginal birth (IVB) can be maternal, such as no progress of cervical dilatation and fetal descent or ineffective maternal efforts, and/or fetal such as an abnormal fetal heart rate. Even if IVB often seems to be preferable to caesarean birth for indications in the second stage of labor [1], in order to preserve the subsequent obstetric prognosis of parturients [2, 3]. However, like caesarean section, it is not devoid of risks and complications [4, 5], in order to provide high quality care it is necessary to be able to analyze this common obstetric practice since it concerns nearly one parturient in eight in France (12%) and like caesarean birth is considered as an operative birth.

To date and to our knowledge, however, there are few recognized tools for the evaluation of practices concerning IVB. Our objective was to create a tool for evaluating IVB practices based on the same principles as Robson's classification of caesarean birth delivery [6]. This new tool would classify parturients according to their obstetric profile in terms of only five parameters, facilitating analysis of IVB practices in each maternity unit, as well as geographical and temporal comparisons.

## Methods

Our classification, which we have called “the Isère classification” (after the county where we work in south-eastern France) is based on predefined obstetric criteria. Our initial criteria were that must be simple to use, robust and require only data that are readily available to the obstetrician in the birthing room. It must be reproducible with a very low rate of inter-observer variation. The number of groups must be sufficient to differentiate the situations encountered in routine practice but sufficiently limited so as not to lose sight of the whole. The order in which the questions are presented and the relationships between the groups are also important allowing an intuitive use and a quick classification of each case. Groups must be fully inclusive, meaning that each woman can be included in one of the groups; and mutually exclusive, which means that each woman can belong to only one group. The different groups in the classification should reflect, as far as possible, the situations most relevant to routine clinical practice.

To meet these requirements, we proposed a series of questions to be posed to the attending obstetrician or midwife, in a predefined order. Only simple yes/no answers were needed before moving to the next question. The groups and questions we initially proposed concerned the multiplicity of the pregnancy, the presentation of the fetus(es), gestational age (GA), type of any previous childbirth (vaginal or cesarian), whether labor was induced or not, and scarred uterus. We aimed for the minimum number of questions that would allow us to form a reasonable number of well-defined groups in the new classification.

We refined and validated our classification using the Delphi method [7]. This is an interactive technique that makes it possible to highlight differences and convergences in opinions and obtain a consensus from a group of experts about the proposed classification. A Delphi study is conducted with a group of people considered to have expertise (both professional and based on experience) in the field, in our case obstetrics. For our classification, an email invitation was sent to 14 national experts. They were all active practitioners in the field and represented the whole of France. Those who responded to the invitation and agreed to participate in all phases of the Delphi process provided written and informed consent to participate and were included in the expert panel. Panel members were blinded to the identities of the other experts. The experts filled out all questionnaires anonymously and then received feedback including answers from all the other experts in the panel. At each round, experts could add comments or suggestions. This process was repeated until the range of expert responses narrowed enough to build a consensus or near-consensus on some or all points.

In the first round, we asked the experts to fill a questionnaire on clinical relevance of each proposed group of our draft classification using a Likert scale with five response options ("not at all relevant", "not very relevant", “no opinion”, “fairly relevant” and “very relevant”). They were also asked to explain their choice when their answer was “not at all relevant” or “not very relevant”. Then, modifications were made to the proposed groups according to the answers. In the second round the revised classification and questionnaire was submitted to the panel along with explanations. Each group of our classification was validated if more than half of the experts considered it to be "fairly relevant" or "very relevant".

Lastly, we conducted a retrospective study to describe the IVB practices in our French level III maternity unit in 2021 (over the whole year), using the new classification.

## Results

All 14 experts we had contacted agreed to participate in the expert panel. Consensus was quickly obtained for each of the seven groups after only two rounds. After the 1st round, on the recommendation of the experts we modified the title of groups 1 to 3 by removing the mention “scarred uterus included” because the characteristics (multiplicity, presentation and gestational age) are sufficient to classify them. We also changed the numbering of the groups to make our classification easier to use. These modifications were validated during the second round, accompanied by explanations.

The overall consensus regarding the proposed classification was good. Of the seven proposed groups two were deemed relevant by all 14 experts, two by 13 experts, and three by 12 experts. Thus, all groups could be validated as more than half of the experts judged each one to be "fairly relevant" or "very relevant". No group was deemed to be “not at all relevant”.

**Fig. 1:**
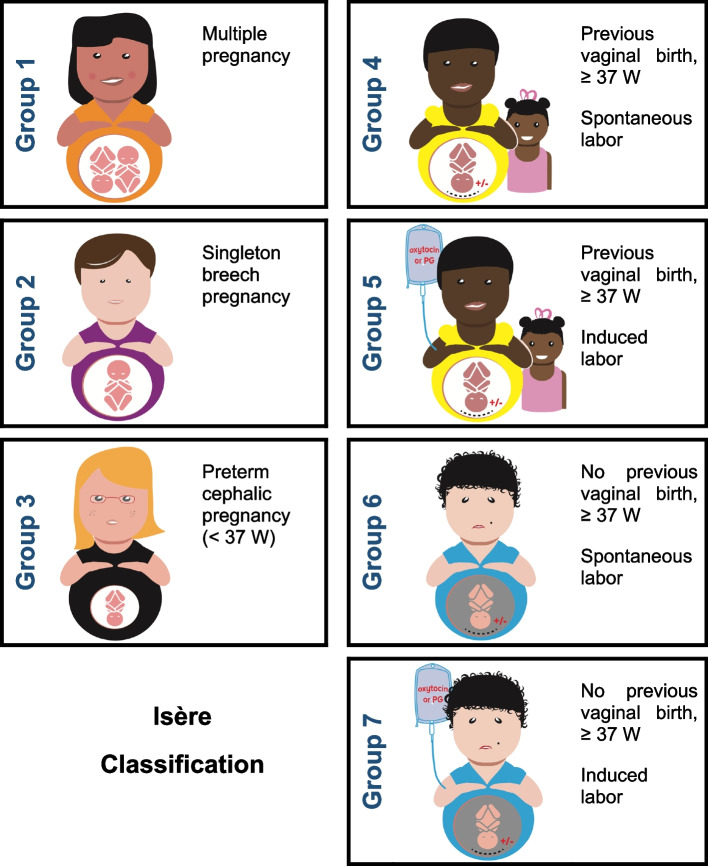
The 7 groups of the “Isère classification” for instrumental vaginal births. Groups 4 and 5 are women with full-term pregnancies who have already given birth vaginally (scarred uterus included); groups 6 and 7 are women who have never given birth vaginally (scarred uterus included). Among groups 4 to 7, groups 4 and 6 are women with spontaneous labor and groups 5 and 7 had undergone induction of labor

Our final classification is based on five obstetric criteria present in all medical records: 1) number of fetuses (singleton or multiple pregnancy), 2) fetal presentation (cephalic or breech), 3) gestational age in weeks of amenorrhea (< or ≥ at 37 GA), 4) any previous history or not of vaginal birth, and 5) the mode of entry into labor. It is made up of seven groups deemed clinically relevant (Fig. 1).

Missing data has led some users of the Robson classification to create a category ‘‘99’’ for these women. We believe this suggestion is very relevant and is why we propose the addition of this “99” group to the Isère classification to make it completely ‘‘totally inclusive’’. The size of the group ‘‘99’’ can be useful to audit the quality of the data. This additional group includes, apart from unclassifiable women, also women with fetal deaths *in-utero* and medical terminations of pregnancy (therapeutic abortion).

- Group 1: Multiple pregnancy
- Group 2: Singleton in breech presentation
- Group 3: Singleton in cephalic presentation at GA < 37 weeks
- Group 4: Singleton in cephalic presentation at GA ≥ 37 weeks, with a history of at least one vaginal birth, spontaneous labor (scarred uterus included)
- Group 5: Singleton in cephalic presentation at GA ≥ 37 weeks, with a history of at least one vaginal birth, induced labor (scarred uterus included)
- Group 6: Singleton in cephalic presentation at GA ≥ 37 weeks, without previous vaginal birth, spontaneous labor (scarred uterus included)
- Group 7: Singleton in cephalic presentation at GA ≥ 37 weeks, without previous vaginal birth, induced labor (scarred uterus included)
- Group 99: *In-utero* fetal death*,* medical termination of pregnancy (therapeutic abortion), unclassifiable women (missing data)

**Fig. 2:**
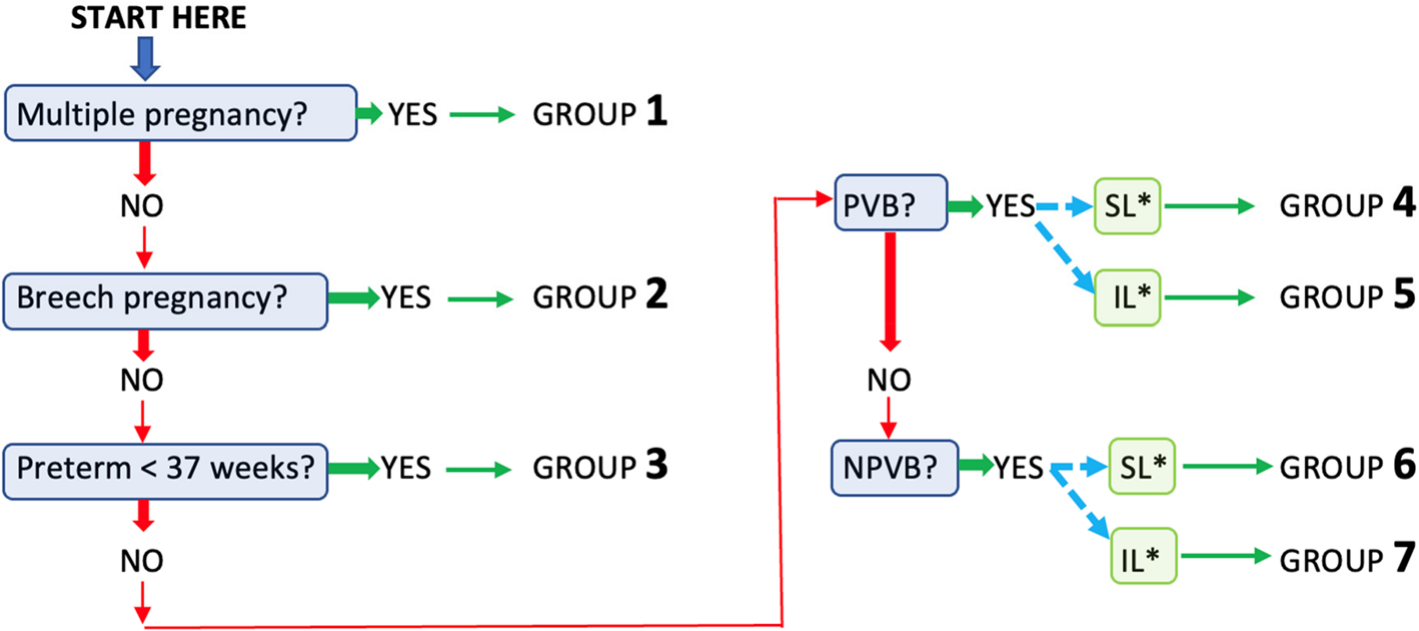
Sequence of the 5 questions allowing each woman to be classified into one of the classification groups (PVB = prior vaginal birth; NPVB = no prior vaginal birth; SL = spontaneous labor; IL = induced labor)

In order to classify each woman into the appropriate group, only five consecutive questions are needed (Fig. 2). The numbering of the groups was defined according to the order of the questions. For each question, if the answer is positive, the woman is placed in the corresponding group. Conversely, if the answer is negative, the process is continued until a positive answer is given. For groups 4 to 7, the final question concerns the mode of entry into labor, spontaneous or induced.

While a multiple pregnancy (group 1) represents only a small proportion of women giving birth, medicalized childbirth, whether by cesarean or by vaginal birth, is very frequent in France for this group.

Breech presentation of a singleton (group 2) represents a small part of IVBs, but because of its particularity it cannot be included in another group (forceps or spatulas are usually used for the after-coming head). 

The specificities of singleton birth in cephalic presentation at < 37 weeks GA (group 3) justifies the creation of a group of its own.

The birth of a singleton in cephalic presentation at GA ≥ 37 weeks (groups 4 to 7) account for the majority of IVBs. We considered it useful to classify these women according to whether or not they had previously given birth by vaginal delivery and not according to their parity, and then to subdivide them according to their mode of entry into labor.

The different groups in our classification reflect the most frequent situations seen in clinical practice with regard to IVBs and account for a significant proportion of IVBs in most maternity units. However, this first classification does not address the problems particular to each group. For this, intra-group analyzes need to be made according to obstetrical criteria such as a history of scarred uterus, the sequential use of instruments, the indication for IVB, the fetal position (rearward or forward), whether analgesia is used during labor, fetal macrosomia, etc.

Our retrospective study of IVB practices over a one-year period (year 2021) in our level III French maternity unit (competent for the management of high-risk pregnancies) found a IVB rate of 17.5% (*n* = 441) for a caesarean section rate of 19.7%. Groups 6 and 7 were predominant in this year of IVB practice, accounting for 70% of IVBs (54% and 16% respectively), and groups 4 and 5 included almost 19% of parturients. No patients were included in the "99" group.

## Discussion

Instrumental vaginal birth is frequent in France and in many other European countries [8–10]. It is often preferred to cesarean delivery for indications in the 2nd stage of labor, but it is not devoid of risks and complications depending on the obstetric prognosis of parturients. A large-scale European study [8] showed that IVB rates varied greatly from country to country and were not always correlated with caesarean section rates within the same country. In metropolitan France, the IVB rate has been stable since 1998 at around 12% [9]. A survey of practices in Europe in 2019 [10] showed large disparities with the lowest IVB rate at 1.4% in Croatia and the highest rate in Spain at 14.4%. This ratio of 1 to 10 is incomprehensible in itself and a more detailed evaluation of practices with dedicated tools seems necessary to improve maternity care of mothers and newborns. To our knowledge, there was no recognized and widely used evaluation tool for this practice described in the literature. Some authors have analyzed the rate of IVBs compared with caesarean section rates, attempting to explain this rate but without using a tool to classify IVBs [11, 12]. They suggested that calculating the ratio in different institutions could help with the analysis of obstetric practices and might lead to a reduction in unnecessary major surgery.

In France, the high rate of operative vaginal birth, involving nearly one in eight parturients justifies the creation of a classification system to monitor and compare IVB practices within and among institutions and among different populations, to analyze trends over time, as well as to compare maternal and perinatal outcomes. While the overall IVB rate in France is acceptable compared to international standards, it is imperative that health systems and facilities are aware of some specific subgroups that could benefit from improvements in the quality and appropriateness of care.

Another interest of our classification is to facilitate evaluations of practices over short periods of time. It could be used in the analysis of the impact of measures implemented to improve the quality of care or of new recommendations for clinical practice. O'Leary et al. [13] suggested that the Robson classification of caesarean section should be used to classify parturients who received instrumental assistance. However, some of Robson’s groups are not applicable to vaginal delivery (such as groups 2b and 4b that are patients whose caesarean section is performed before labor begins, and group 9 (transverse fetal presentation) where vaginal delivery is not possible. Nevertheless, we were influenced by Robson's classification [14].

We propose a classification for women requiring IVB made up of seven mutually exclusive and totally inclusive groups based on five readily available and reliably collected variables. These groups are easy to use, clinically relevant and easy to implement at local, regional, national or even international level. All groups can be further subdivided in order to determine common denominators during the analysis of the results and thus better target the populations at risk of IVB.

Using the same approach as for the recently published Grenoble classification of induced labor [15], our IVB classification was submitted to 14 national experts using a Delphi method. This qualitative method reflects the subjective and consensual opinions of a group of experts [16]. It makes it possible to generate a reasoned consensus opinion that can be used to legitimize the choices made when creating the classification. In the first-round we immediately proposed a classification with the groups defined in advance. An alternative approach would have been to question the experts about their proposals for the initial obstetrical criteria io be used; gradually creating the different groups.

While the randomized controlled study by Grobman et al. (2018) [17] found no statistically significant differences in resort to IVB whether or not labor was induced (7.3% if induced vs. 8.5% if spontaneous, *p* = 0.07), it should be remembered that induction practices in certain European countries are very different from those in France (with a rate of at least 15% in nulliparous women). In addition, this study only concerned low-risk nulliparous women, whereas multiparous women represent a significant proportion of births in France (58.6% according to the 2021 National Perinatal Survey [8]).

After searching the literature, we found no convincing tool to assess the impact of induction of labor on the use of IVB. This is why it seemed judicious to decern between the types of labor in parturients with or without previous vaginal delivery. In the study by O'Leary et al. [13], a higher rate of IVB in their population of nulliparous women with induced labor was observed as compared to spontaneous labor. This increased risk could be related to the reason for induction of labor. However, it seemed important to be able to objectify it in order to best advise patients, particularly as the onset of labor plays a key part in the management of childbirth.

We chose not to reason in terms of the overall parity of the woman but according to a precedent of at least one vaginal birth. This made it possible to group together nulliparous women with multiparous women who had never given birth vaginally (history of 1 or 2 caesarean sections in their previous births); and to group-together women who already had a history of vaginal birth, whether or not they had a scarred uterus. The latter represented 19% of the women included in our retrospective study of practices in 2021.

In order to limit interpretation bias the experts recommended no to take into account the medical indication for IVB, although this could be included in more detailed studies.

The failure of an attempt at instrumental vaginal birth was not included in our classification since it generally results in caesarean section. Nevertheless, every establishment should be aware of the failure rate of the instrumental vaginal route. An evaluation of IVB practices using our classification could lead to actions aimed at improving practices, for some if not all groups. The widespread use of this classification might make it easier to analyze IVB practices at local, regional, national or even international level. It should assist in improving our ability to compare relatively obstetrically homogeneous populations of women and thus contribute to improving the quality of maternity care.

The retrospective study of practices in our maternity unit enabled us to highlight groups with a relatively high proportion of IVBs. These were mainly women with a singleton pregnancy in cephalic presentation at ≥ 37 weeks GA, without previous vaginal birth, with spontaneous labor (scarred uterus included) (group 6).

In the future, we plan to continue more detailed analysis of our own practices in order to provide high quality maternity care and to monitor its evolution over time. The comparison of our practices with those of other maternity units using the Isère classification would be of great interest.

Each maternity unit, network, region or country may have different expectations regarding IVB practices depending on the extent of use. For maternities whose IVB rate is higher than the national rate, the objective could be to reduce it by increasing the rate of spontaneous vaginal births. One of the avenues for improvement to promote spontaneous birth would be to consider a longer duration of the 2nd stage of labor, and to allow an extension of the duration of expulsive efforts in certain groups. Appropriate use of a classification for IVBs could help improve practices of instrumental vaginal birth and coupled with the Robson classification, it would clearly be useful in describing the practices of all operative births in each maternity unit and beyond.

## Conclusion

We have created a tool for classifying women in childbirth according to their obstetric circumstances to facilitate the evaluation of instrumental vaginal birth practices. This will make it easier not only to analyze practices within each maternity unit but also to be able to make geographical and temporal comparisons. The tool is a simple, robust classification, comprising seven groups based on five obstetric criteria easily available in each woman's medical file.

## Data Availability

The de-identified datasets used and analyzed during the study are available from the corresponding author on reasonable request.

## Abbreviations

GA: Gestational age
IVB: Instrumental vaginal birth
IL: Induced labor
PVB: Prior vaginal birth
NPVB: No prior vaginal birth
SL: Spontaneous labor
